# Supplementary material for: Proportions of Polyunsaturated Fatty Acids in Umbilical Cord Blood at Birth Are Related to Atopic Eczema Development in the First Year of Life
Source: Nutrients. 2021 Oct 25;13(11):3779. doi: 10.3390/nu13113779 (PMC8624970; doi:10.3390/nu13113779)
Supplement: Supplementary file 1 [file nutrients-13-03779-s001.zip › Supplementary material.pdf]

nutrients

Supplementary material for

Proportions of Polyunsaturated Fatty Acids in Umbilical Cord Blood at Birth Are Related to Atopic Eczema Development in the First Year of Life

Malin Barman [1,2,*], Mia Stråvik [1], Karin Broberg [2,3], Anna Sandin [4], Agnes E. Wold [5] and Ann-Sofie Sandberg [1]

1 Food and Nutrition Science, Department of Biology and Biological Engineering, Chalmers University of Technology, Sweden; malin.barman@chalmers.se (M.B.), mia.stravik@chalmers.se (M.S.), ann-sofie.sandberg@chalmers.se (A-S.S.)
2 Institute of Environmental Medicine, Karolinska Institutet, Stockholm, Sweden
3 Department of Laboratory Medicine, Occupational and Environmental Medicine, Lund University, Lund, Sweden;
4 Department of Clinical Science, Pediatrics, Sunderby Research Unit, Umeå University, Sweden; anna.sandin@umu.se,
5 Institute of Biomedicine, Department of Infectious Diseases, The Sahlgrenska Academy, University of Gothenburg, Sweden; agnes.wold@microbio.gu.se
* Correspondence: malin.barman@chalmers.se; Tel.: +4631-7723811

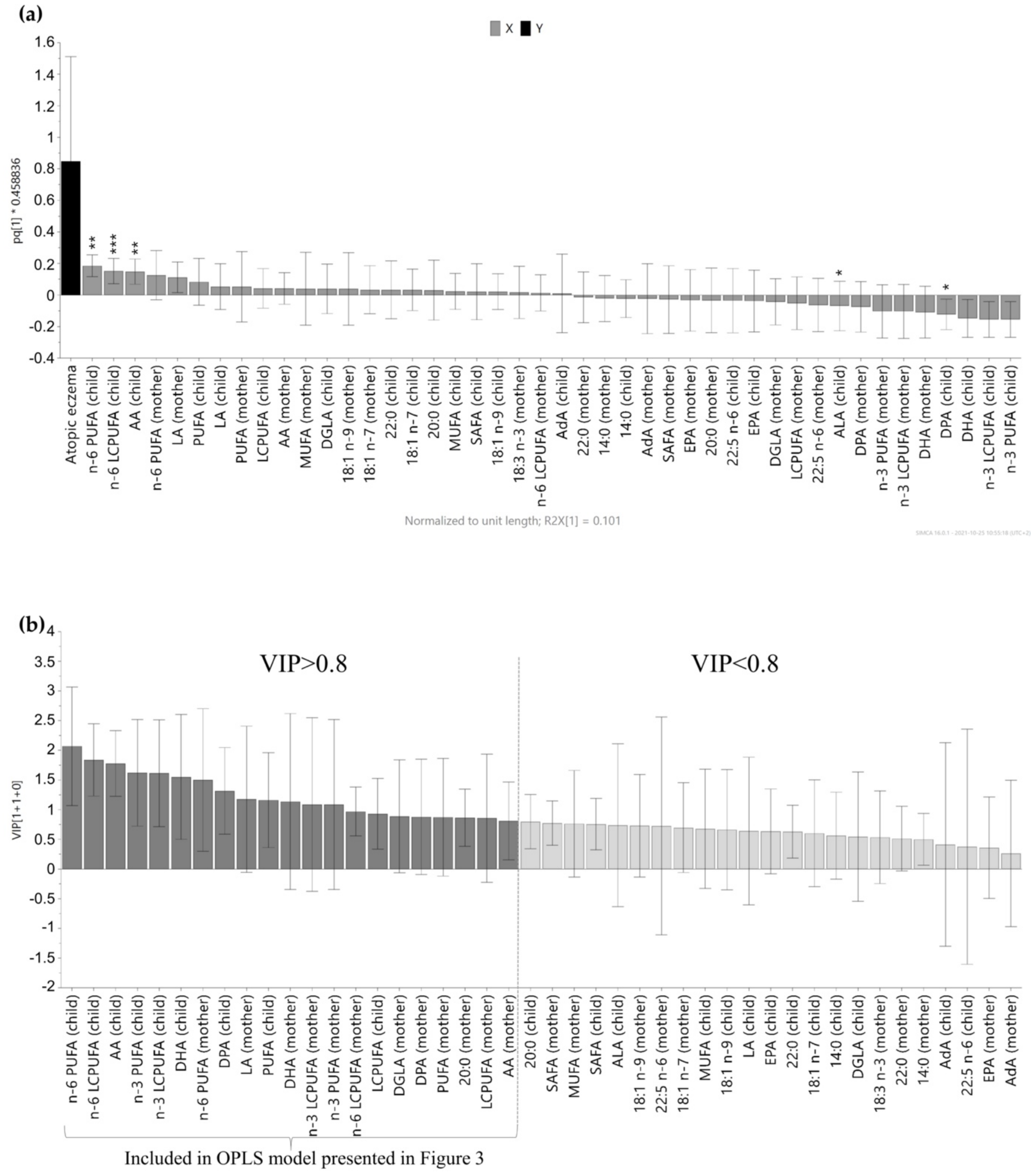

Supplementary Figure S1. Orthogonal partial least square (OPLS) model of the associations between atopic eczema at 12 months of age and fatty acids in maternal and cord plasma phospholipids. **(a)** Loading column plot of all fatty acids in relation to atopic eczema. **(b)** Variable of importance (VIP) plot. Associations that were significant in univariate analyses (Mann–Whitney *U*-test) are indicated with an asterisk: * $p < 0.05$, ** $p < 0.01$, *** $p < 0.001$.

Supplementary Table S1. Primer pairs for detecting variations in the filaggrin gene.

Mutation	rs-ID	Primer pairs (5′–3′)
R501X	rs61816761	Forward AGCACTGGAGGAAGACAAGGATC<br>Reverse ACCCTCTTGGGACGCTGAAT′
R2477X	rs138726443	Forward CACGTGGCCGGTCAGCA<br>Reverse TCCTGACCCTCTTGGGACGT
S3247X	rs150597413	Forward CCAGAAACCATCGTGGATCTG<br>Reverse TGCCTGATTGTCTGGAGCG
2282del4	rs41370446	Forward 1 TCCCGCCACCAGCTCC<br>Forward 2 CCACTGACAGTGAGGGACATTCA<br>Reverse GGTGGCTCTGCTGATGGTGA
**CNV**[1]	**rs-ID**	**Primer pairs**
12 repeat	rs12730241	Predesigned SNP genotyping assay by Applied Biosystems

[1] Copy Number Variation

nutrients MDPI

Supplementary Table S2. Median (25th-75th percentile) fatty acid proportions of phospholipids in maternal and infant plasma at birth.

	Median (25th-75th percentile)			Correlation between infant and maternal plasma[2]	
	**Infant plasma**	**Maternal plasma**	**P[1]**	**Rho**	**P**
**Saturated**					
14:0	0.74 (0.59-0.87)	0.82 (0.67-0.94)	<0.001	0.40	<0.001
20:0	0.88 (0.77-1.02)	0.51 (0.44-0.59)	<0.001	0.50	<0.001
22:0	1.6 (1.4-1.8)	1.2 (1.0-1.4)	<0.001	0.35	<0.001
Total	3.2 (2.8-3.8)	2.6 (2.3-2.9)	<0.001	0.40	<0.001
**Monounsaturated**					
18:1 n-7	2.6 (2.3-3.0)	1.5 (1.3-1.6)	<0.001	0.43	<0.001
18:1 n-9	12 (10-13)	16 (14-17)	<0.001	0.39	<0.001
Total	14 (12-16)	17 (16-19)	<0.001	0.43	<0.001
**n-6 Fatty acids**					
18:2 n-6 (LA)	14 (13-16)	38 (36-41)	<0.001	0.50	<0.001
20:3 n-6 (DGLA)	12 (10-13)	7.6 (6.8-8.9)	<0.001	0.52	<0.001
20:4 n-6 (AA)	37 (34-40)	19 (17-21)	<0.001	0.58	<0.001
22:4 n-6	1.3 (1.1-1.5)	0.55 (0.46-0.64)	<0.001	0.44	<0.001
22:5 n-6	0.99 (0.79-1.3)	0.55 (0.41-0.74)	<0.001	0.42	<0.001
Total long-chain	52 (48-54)	28 (26-30)	<0.001	0.57	<0.001
Total	66 (63-69)	66 (64-69)	0.171	0.58	<0.001
**n-3 Fatty acids**					
18:3 n-3 (ALA)	0.04 (0.03-0.05)	0.18 (0.14-0.23)	<0.001	0.13	0.071
20:5 n-3 (EPA)	0.91 (0.72-1.2)	1.4 (1.1-1.8)	<0.001	0.61	<0.001
22:5 n-3 (DPA)	1.1 (0.87-1.4)	1.3 (1.0-1.5)	<0.001	0.41	<0.001
22:6 n-3 (DHA)	14 (12-17)	11 (9.3-13)	<0.001	0.48	<0.001
Total long-chain	16 (14-19)	14 (12-16)	<0.001	0.52	<0.001
Total	16 (14-20)	14 (12-17)	<0.001	0.53	<0.001

[1] Difference between maternal and infant samples were analyzed with Wilcoxon signed-rank test.

[2] Associations between maternal and infant fatty acid proportions were tested with Spearman correlation test.

nutrients MDPI

Supplementary Table S3. Relative proportions of phospholipids in cord serum at birth in children with atopic eczema compared to non-allergic children.

	Atopic eczema, n=14		Non-allergic, n=192		
Cord plasma fatty acids	Median	(25th-75th)	Median	(25th-75th)	P-value
**Saturated**					
14:0	0.71	(0.58-0.86)	0.76	(0.60-0.89)	0.667
20:0	0.86	(0.68-0.99)	0.88	(0.77-1.0)	0.498
22:0	1.5	(1.4-1.8)	1.6	(1.4-1.9)	0.540
Total	3.2	(2.7-3.7)	3.3	(2.8-3.8)	0.439
**Monounsaturated**					
18:1 n-7	12	(9.8-14)	12	(10-14)	0.664
18:1 n-9	2.6	(2.2-2.9)	2.6	(2.3-3.0)	0.528
Total	14	(12-17)	14	(12-17)	0.664
**n-6 Fatty acids**					
18:2 n-6 (LA)	15	(13-17)	14	(13-16)	0.763
20:3 n-6 (DGLA)	11	(9.9-12)	12	(10-13)	0.351
20:4 n-6 (AA)	40	(38-44)	37	(34-40)	**0.002**
22:4 n-6	1.3	(1.1-1.6)	1.3	(1.1-1.5)	0.938
22:5 n-6	1.0	(0.62-1.2)	0.98	(0.79-1.3)	0.725
Total long-chain	54	(53-57)	51	(48-54)	**0.001**
Total	69	(67-72)	66	(63-68)	**0.002**
**n-3 Fatty acids**					
18:3 n-3 (ALA)	0.03	(0.02-0.04)	0.04	(0.03-0.05)	**0.020**
20:5 n-3 (EPA)	0.74	(0.58-1.2)	0.94	(0.74-1.2)	0.133
22:5 n-3 (DPA)	0.95	(0.64-1.07)	1.1	(0.86-1.4)	**0.026**
22:6 n-3 (DHA)	12	(9.9-16)	14	(11-17)	0.192
Total long-chain	14	(11-18)	16	(14-19)	0.126
Total	14	(11-18)	16	(14-19)	0.125
**Polyunsaturated**					
Total long-chain	70	(66-72)	69	(65-72)	0.272
Total	84	(82-87)	83	(80-85)	0.116

p-value obtained with Mann-Whitney U test.

Supplementary Table S4. Relative proportions of phospholipids in **maternal** serum at birth in mothers to children with atopic eczema compared to mothers to non-allergic children

	Atopic eczema, n=13		Non-allergic, n=180		
Maternal plasma fatty acids	Median	(25th-75th)	Median	(25th-75th)	P-value
**Saturated**					
14:0	0.80	(0.63-0.95)	0.82	(0.67-0.95)	0.685
20:0	0.48	(0.46-0.57)	0.51	(0.44-0.59)	0.507
22:0	1.2	(1.0-1.3)	1.2	(1.0-1.4)	0.336
Total	2.6	(2.2-2.7)	2.6	(2.3-2.9)	0.329
**Monounsaturated**					
18:1 n-7	15	(15-18)	16	(14-17)	0.750
18:1 n-9	1.5	(1.4-1.6)	1.4	(1.3-1.6)	0.447
Total	17	(16-19)	17	(16-19)	0.793
**n-6 Fatty acids**					
18:2 n-6 (LA)	39	(36-44)	38	(35-41)	0.494
20:3 n-6 (DGLA)	7.8	(6.3-8.3)	7.6	(6.8-8.9)	0.453
20:4 n-6 (AA)	21	(18-23)	19	(17-21)	0.074
22:4 n-6	0.52	(0.35-0.70)	0.55	(0.46-0.64)	0.723
22:5 n-6	0.56	(0.37-0.74)	0.55	(0.41-0.74)	0.988
Total long-chain	30	(28-31)	28	(26-30)	0.160
Total	68	(66-71)	66	(64-68)	0.119
**n-3 Fatty acids**					
18:3 n-3 (ALA)	0.21	(0.14-0.22)	0.18	(0.14-0.23)	0.688
20:5 n-3 (EPA)	1.3	(1.1-2.0)	1.4	(1.1-1.8)	0.738
22:5 n-3 (DPA)	1.2	(0.95-1.7)	1.3	(1.1-1.5)	0.707
22:6 n-3 (DHA)	11	(8.8-12)	11	(9.4-13)	0.212
Total long-chain	13	(11-15)	14	(12-17)	0.349
Total	13	(11-15)	14	(12-17)	0.352
**Polyunsaturated**					
Total long-chain	42	(41-43)	42	(38-46)	0.975
Total	81	(80-84)	80	(79-82)	0.170

p-value obtained with Mann-Whitney U test.
